# Impacts of Dual-Income Household Rate on Suicide Mortalities in Japan

**DOI:** 10.3390/ijerph18115670

**Published:** 2021-05-25

**Authors:** Misaki Nakamoto, Takatoshi Nakagawa, Masahiko Murata, Motohiro Okada

**Affiliations:** 1 Department of Neuropsychiatry, Division of Neuroscience, Graduate School of Medicine, Mie University, Tsu, Mie 514-8507, Japan; m-nakamoto@clin.medic.mie-u.ac.jp (M.N.); t-nakagawa-4823@med.mie-u.ac.jp (T.N.); 2 Department of Psychiatry, National Hospital Organization Sakakibara Hospital, 777 Sakakibara, Tsu, Mie 514-1292, Japan; muyuhton@gmail.com

**Keywords:** suicide mortality, Japan, prefecture, dual-income household, life–work balance, family–work balance

## Abstract

To explore impact of enhancing social advancement of females in Japan, this study determined the effects of the dual-income household rate on suicide mortalities disaggregated by attributes of gender, age, and motives between 2009 and 2017 in Japan. This study analysed impact of dual-income household rate, other household-related factors (savings, liabilities and yearly incomes per household, minors and elderly rate per household), and social/employment factors (complete unemployment rate, employment rate, temporary male and female employment rates and certification rate of long-term care insurance) on suicide mortalities disaggregated by attributes of gender, age, and motives using hierarchical linear-regression model. Dual-income household rate was significantly/negatively related to suicide mortality of the working-age female population, but significantly/positively related to that of the elderly female population. Suicide mortalities of the working-age male population and the elderly male population were significantly/positively related to dual-income household rate. Male suicide mortalities caused by family-, health-, economy- and employment-related motives were significantly/positively related to dual-income household rate; however, the dual-income household rate was significantly/positively related to female suicide mortalities caused by family-, health-, economy- and school-related motives, but significantly/negatively related to suicide mortalities caused by romance-related motives. Dual-income households suppress social-isolation and develop economical/psychological independence of females, leading to reduced suicide mortality in working-age females. However, elderly and school-age populations, who are supported by the working-age female, suffer from isolation. Working-age males also suffer from inability to adapt from the traditional concept of work–life and work–family balances to the novel work–family balance concept adapted to dual-income households. These results suggest occurrence of new social/family problems in the 21st century due to vulnerability of traditional Japanese culture and life–working–family balance concepts as well as novel sociofamilial disturbances induced by declining birth rate and ageing population in Japan.

## 1. Introduction

In the past decade, despite severe socioeconomic decline, there has been an improvement in the suicide mortality rate in Japan [1,2,3]. Several recent studies have reported the impact of governmental financial support on improving suicide mortality in Japan, Europe and the US. The enhancement of labour market programmes supported by governmental finances in these regions may have contributed to the improvement in the suicide mortality rate due to socioeconomic disabilities [1,2,4,5,6]. Indeed, an increase in participation in the Supplemental Nutrition Assistance Program (SNAP) in the US has been related to reduced suicide mortalities [7]. It has been reported that the governmental financial support provided to regional suicide prevention programmes played an important role in improving suicide mortality in Japan [1,2,3]; however, these studies indicated that these programmes mainly contributed to reducing male suicide mortality, and had a limited contribution to female suicide mortality [1,2,3]. Various studies, which explored this “gender paradox of suicide”, reported that males more frequently die by suicide than females, except in some Asia countries [1,2,3,8,9,10,11,12]. Based on these findings, we hypothesised that inadequate social/welfare support resources for childcare and caring for infirm family members in Japan impede social advancement of females, resulting in a lack of improvement in the social isolation of females in Japan [1,2,3], since the primary responsibility for the care of children and infirm family members that is undertaken by Japanese females was found to be one of the major suicide motives among females [3,13].

Considering the labour shortage and financial problems due to the declining birth rate and ageing population in Japan [14], the social advancement of females contributes to compensating for labour shortages and improving female quality of life and suicide mortality in Japan. In other words, the enhancement of the economic and social participation and contribution of women contributes to the socioeconomic development in Japan. Against these severe socioeconomic conditions and public health crisis in Japan, according to the popular concept of work–family culture which includes ‘the shared assumptions, beliefs, and values regarding the extent to which an organisation supports and values the integration of employee’s work and family lives’ [15], the Gender Equality Bureau Cabinet Office enacted the ‘Fifth Basic Plan for Gender Equality’ to promote the social participation and contribution of females in Japan [16]. The concept of work–family culture is composed of three major factors: work–family organisational support, career consequences related to the use of work–family programmes, and workplace expectations hindering the fulfilment of the family role [15]. However, along with these advantages regarding the promotion of social advancement of females, dual-income households suffer from several issues in terms of conciliation among the working environment, the family context and the management of children and infirm family members [17]. Therefore, Japanese society as well as individuals must endeavour to adapt from the traditional work–life balance concept to a novel work–life balance model, which incorporates the declining birth rate and the ageing population. In other words, we should assume that the rapid promotion of women’s social participation and contribution generates disturbances via progression of vulnerability of the immature social/welfare system associated with work–family balance in Japan; however, the impact of social participation and contribution of females on suicide mortality and familial psychodynamics in Japan remains to be clarified.

Thus, based on the background of Japanese society, to clarify the impact of social/familial situations on suicide mortality in Japan, the present study determined the effects of the dual-income household rate, other familial factors such as familial savings, liabilities, yearly incomes, minors and elderly rates per household, and social factors such as complete unemployment rate, employment rate, temporary employment rates and certification of long-term care insurance rate on suicide mortalities disaggregated by region (prefecture), gender, age, and motives using the hierarchical linear regression model with the compensation of fixed-effect regression and robust standard error.

## 2. Materials and Methods

### 2.1. Dependent Variables

The data of suicide victims in all 47 prefectures in Japan between 2009 and 2017 were obtained from the national governmental database, the Basic Data on Suicide in the Region (BDSR) of the Ministry of Health, Labour and Welfare (MHLW) [18]. The statistics data in BDSR were published the number of suicide individuals identified by the regional police stations which investigate using the evidence, suicide note and documentation, such as medical certificate, clinical recording and testimony [3,13,18]. BDSR showed the annual suicide numbers caused by six major motives: family-, health-, economy-, employment-, romance- and school-related motives disaggregated by gender [18]. BDSR also classified the annual suicide data based on the following age groups and gender: those younger than 20 years old (10s), 20–29 (20s), 30–39 (30s), 40–49 (40s), 50–59 (50s), 60–69 (60s), 70–79 (70s) and over 80 years old (80s) [18]. Annual prefectural suicide mortalities were calculated by dividing the suicide mortalities per prefecture by the prefectural population (denominator) of the same years obtained from the Regional Statistics Database: System of Social and Demographic Statistics of the Statistics Bureau of the Ministry of Internal Affairs and Communications (SBMIAC) [19]. To eliminate small prefectural population artefacts, the prefectural suicide mortalities were calculated by using the empirical Bayes (EB) standardised mobile ratio method by using the EB estimator for the Poisson/gamma model (Version 2.1) (National Institute of Public Health, Wako, Japan) (https://www.niph.go.jp/soshiki/gijutsu/download/ebpoig/index_j.html accessed on 1 March 2021) [20]. Annual standardised death rates for suicide mortalities (SDR) of males, females and males + females were calculated based on the Japanese age-dependent population composition in 2009 for males and females. The WHO world standard population model was not considered [21], since the age distribution in Japan is significantly different from that in the WHO world standard population model [1].

### 2.2. Independent Variable and Covariates

The covariates were composed of two types of factors: household structural factors and social/employment factors. Data relating to household structural factors, including household economic factors such as savings, liabilities and yearly incomes per household (million yen), and structural factors such as the rates of minors and the elderly per household (including age groups below 18 years of age and over 65 years of age) per household, were obtained from the Family Income and Expenditure Survey in the Statistics Bureau of the Ministry of Internal Affairs and Communications (SBMIAC) [22]. Data relating to social/employment factors, such as certification rate of long-term care insurance (per 100,000 population), complete unemployment rate, employment rate, and male and female temporary employment rates were obtained from the Family Income and Expenditure Survey (SBMIAC) [22], Labour Force Survey (SBMIAC) [23] and System of Social and Demographic Statistics (SBMIAC) [24], respectively. The targeted independent variable, the dual-income household rate, was derived by using the method of least squares by using BellCurve for Excel (version 3.2) (BellCurve, Tokyo, Japan) [3] from the data obtained from the Employment Status Survey (SBMIAC) [25] and Population Census (SBMIAC) [26].

The BDSR was published in March of the following year [18], whereas the latest data in the Employment Status Survey were published at 2017 [25]. Therefore, the present study analysed using data ranged from 2009 to 2017.

### 2.3. Statistical Analysis

The present study analysed the impact of the dual-income household rate, household structure and social/employment factors on suicide mortality in Japan by using a pooled sectional design with fixed-effects regression for prefectures and years using a hierarchical linear regression model (HLM7, Scientific Software International, Skokie, IL, USA). While the fixed-effects model is not affected by unobserved time-invariants, it can detect time-invariant characteristics, such as aspects of culture that are resistant to change. Additionally, the present study adopted robust standard errors clustered by prefectures to prevent heteroscedasticity and autocorrelation. Initially, to prevent multicollinearity, the present study analysed the variance inflation factor (VIF) using the free statistical software HAD version 17 (Shimizu, H., Kansei Gakuin University, Nishinomiya, Hyogo, Japan) (https://osf.io/32cyp/files/ accessed on 1 March 2021) [3]. Any factors with VIF > 4 were removed from analyses [1,2,3]. A three-step strategy was implemented for data analyses. In the first step, only the correlation between dependent variables and the dual-income household rate was analysed by using hierarchical linear regression model with robust standard errors clustered at the prefectural level using HLM7 (Model_1). In the second step, the household structural and social and employment factors were added to Model_1 (Model_2). To explore the specific impacts of the household situation on suicide mortality of school-age populations, the household structural factors alone were added to Model_1 (Model_3).

## 3. Results

The descriptive statistics of dependent (suicide mortalities) and independent variables are summarised in Supplementary Material Tables S1 and S2.

### 3.1. Effects of Houeshold and Social Factors on SDR between 2009 and 2017

In Model_1, the hierarchical linear regression model detected a significant/positive relationship between the dual-income household rate and SDR of males + females and males, whereas the SDR of females was not related to the dual-income household rate (Table 1). In Model_2, the SDR of males + females, males, and females was significantly/positively related to the dual-income household rate (Table 1). Furthermore, the complete unemployment rate was also significantly/positively related to the SDR of males + females, males, and females (Table 1). In males + females, the yearly income per household and certification rate of long-term care insurance were significantly/negatively related to the SDR of males + females (Table 1). Savings and yearly incomes per household were significantly/negatively related to the SDR of males (Table 1). The elderly rate per household was significantly/negatively related to the SDR of females (Table 1).

### 3.2. Effects of Houeshold and Social and Employment Factors on Suicide Mortalities Caused by Six Major Motives between 2009 and 2017

**Figure 1:**
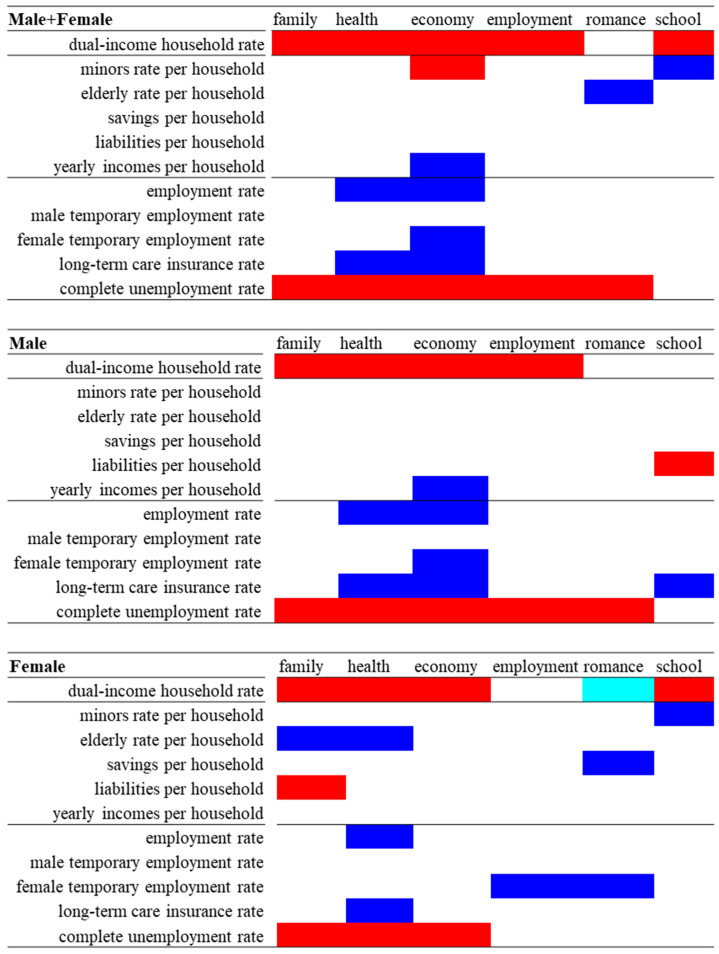
Summary of impacts of dual-income household rate, household structural and social/employment factors on suicide mortalities of Male + Female, Male and Female caused by family-, health-, economy-, employment-, romance- and school-related motives between 2009 and 2017. Blue and red columns indicate significant factors for decreasing and increasing suicide mortalities caused by each motive, respectively, in either Model_2. Light blue column indicates significant factors for decreasing suicide mortalities caused by each motive in Model_1, but not in Model_2.

In Model_1 of both males + females and males, the hierarchical linear regression model detected significant/positive relationship between dual-income household rate and suicide mortality caused by family- and employment-related motives (Figure 1, Supplementary Material Tables S3 and S4). In Model_2, suicide mortalities of males + females and males caused by family-, health-, economy- and employment-related motives were significantly/positively related to the dual-income household rate and complete unemployment rate. Suicide mortalities of males + females and males caused by romance-related motives were significantly and positively related to the complete unemployment rate but were not affected by the dual-income household rate. Neither the dual-income nor the complete unemployment rate was related to male suicide mortality caused by school-related motives. While the suicide mortality of males + females was significantly/positively related to the dual-income household rate, it was not related to the complete unemployment rate. Interestingly, suicide mortalities of males + females and males caused by school-related motives were positively related to the minors rate per household and the certification rate of long-term care insurance, respectively (Figure 1, Supplementary Material Tables S3 and S4). 

In Model_1 of females, while the dual-income household rate was significantly/negatively and significantly/positively related to female suicide mortalities caused by romance- and school-related motives, respectively, no other significant relationship was observed (Figure 1 and Supplementary Material Table S5). In Model_2, while female suicide mortalities caused by romance-related motives were not related to the dual-income household rate, they were significantly/negatively related to the variables of savings per household and female temporary employment rate. Both the dual-income household rate and complete unemployment rate were significantly/positively related to female suicide mortalities caused by family-, health-, and economy-related motives. The rate of the elderly per household was significantly/negatively related to female suicide mortalities caused by family- and health-related motives, and the certification rate of long-term care insurance was significantly/negatively related to female suicide mortality caused by health-related motives. Female suicide mortality caused by school-related motives was significantly/positively and significantly/negatively related to dual-income household rate and minors rate per household, respectively (Figure 1 and Supplementary Material Table S5).

Therefore, the rise in the dual-income household rate indicates an increase in male suicide mortalities caused by family-, health-, economy- and employment-related motives. Conversely, the increase in the dual-income household rate indicates a decrease in female suicide mortalities caused by romance-related motives, but also indicates an increase in female suicide mortalities caused by family-, health-, economy- and school-related motives. These results suggest that the dual-income household rate affects suicide mortality due to gender-related motives and age-specific disturbances associated with familial psychodynamics or the life cycle of suicidal victims.

### 3.3. Effects of Household Structure and Social and Employment Factors on Age-Dependent Suicide Mortalities between 2009 and 2017

**Figure 2:**
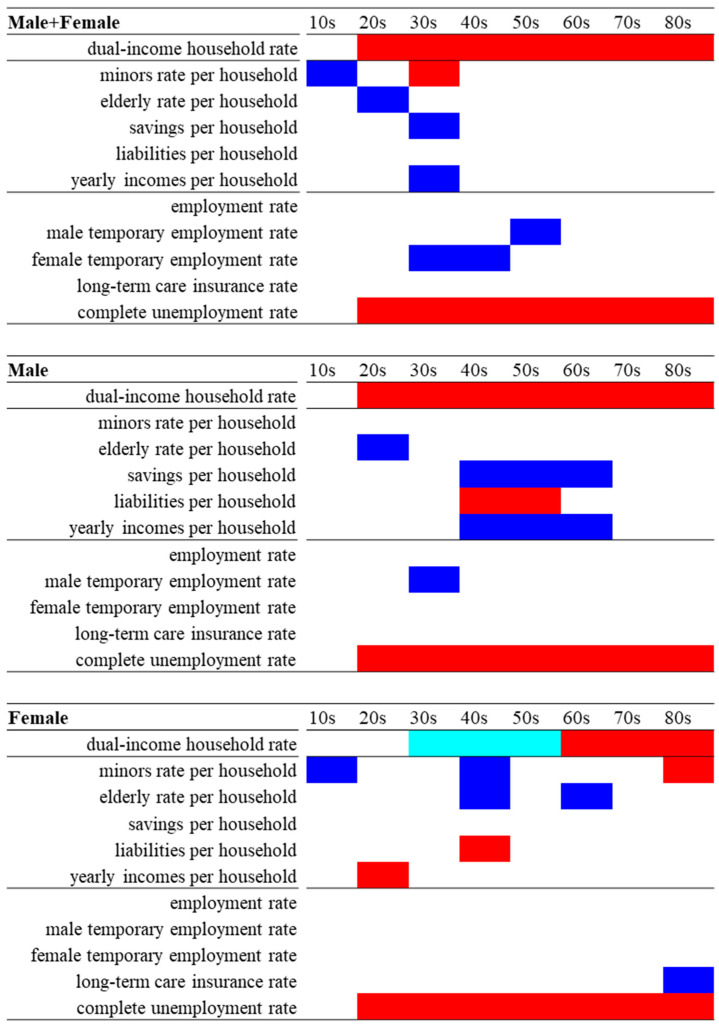
Summary of impacts of dual-income household rate, household structural and social/employment factors on suicide mortalities of Male + Female, Male and Female disaggregated by ages between 2009 and 2017. Blue and red columns indicate significant factors for decreasing and increasing suicide mortalities caused by each motive, respectively, in Model_2. Light blue column indicates significant factors for decreasing suicide mortalities caused by each motive in Model_1, but not in Model_2.

**Figure 3:**
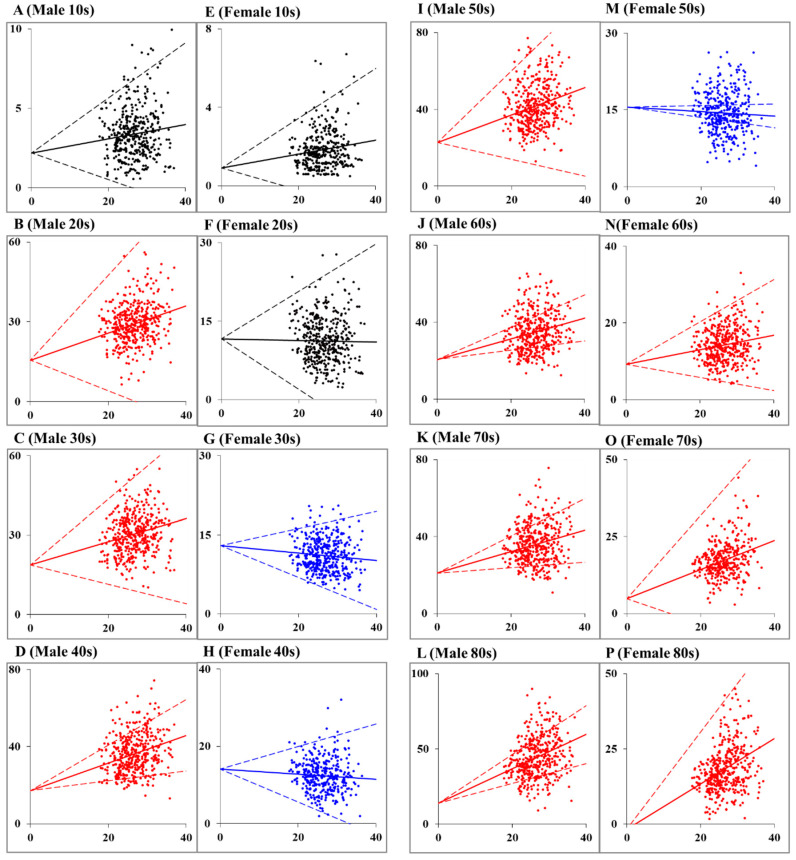
Correlation between dual-income rate and suicide mortalities of Male and Female disaggregated by ages using random-effect analysis of hierarchal linear regression model. Ordinates and abscissas indicate suicide mortality (per 100,000 population) and dual-income household rate (%), respectively. Blue, red and black lines indicate significant decreasing, increasing and no effect on suicide mortalities, respectively. Full and dotted lines indicate estimated random effects regression and standard deviations by hierarchal linear regression model, respectively.

In Model_1 of males + females, the dual-income household rate was significantly/positively related to suicide mortalities of the elderly in their 70s and 80s (Figure 2 and Figure 3, and Supplementary Material Table S6). In Model_1 of males, the dual-income household rate was significantly/positively related to suicide mortalities in 20s, 40s, and 80s. In Model_2 of males + females and males, both the dual-income household rate and complete unemployment rate were significantly/positively related to suicide mortalities in their 20s to 80s, but not to those in their 10s. For males + females in their 10s and 20s, suicide mortality was significantly/negatively related to the rate of minors and elderly per household, respectively. Additionally, suicide mortalities of males + females in their 30s, 40s, and 50s were significantly/negatively related to female and male temporary employment rates, respectively. Male suicide mortalities in their 20s were significantly/negatively related to the rate of the elderly per household, but males’ suicide mortality in 10s was not related to any independent variables. Additionally, suicide mortality of males in their 30s was significantly/negatively related to the male temporary employment rate (Figure 2 and Figure 3, and Supplementary Material Tables S6 and S7).

In Model_1 of females, the dual-income household rate was significantly and positively related to suicide mortalities of females in their 70s and 80s, but significantly/negatively related to suicide mortalities of females in their 30s, 40s and 50s (Figure 2 and Figure 3, and Supplementary Table S8). In Model_2, the negative relationship between the dual-income household rate and suicide mortalities of females in their 30s, 40s, and 50s was abolished, but the suicide mortalities of females in their 60s, 70s and 80s remained related to the dual-income household rate. The complete unemployment rate was significantly/positively related to suicide mortalities of females in their 20–80s. Suicide mortality of females in their 80s was significantly/negatively related to the certification rate of long-term care insurance (Figure 2 and Figure 3, and Supplementary Material Table S8).

Therefore, the rise in the dual-income household rate indicates an increase in suicide mortalities of working-age male and elderly male populations (Figure 2 and Figure 3). On the contrary to males, suicide mortalities of working-age female populations show a decrease with an increase in the dual-income household rate, whereas those of female elderly populations were conversely increased by the rise in the dual-income household rate (Figure 2 and Figure 3). These results also suggest that the dual-income household rate affects suicide mortality due to gender- and age-specific disturbances associated with familial psychodynamics or the life-cycle of suicide victims.

### 3.4. Effects of Household Factors on Suicide Mortalities of School-Age Populations between 2009 and 2017

The analyses of Model_1 and Model_2 showed that suicide mortalities of school-age populations were not sensitive to social/employment factors. Therefore, to explore the specific impacts of household factors on suicide mortalities of school-age populations, the household factors and suicide mortalities of individuals in their 10s and caused by school-related motives were analysed using Model_3 (Table 2). Model_3 could not detect a significant relationship between the suicide mortalities of males in their 10s and household factors, whereas suicide mortalities of males + females and females in their 10s were significantly/negatively related to minors’ rate per household (Table 2). The relationship between males’ suicide mortality caused by school-related motives and household factors was also not observed, whereas both males + females and female suicide mortalities caused by school-related motives were positively and negatively related to the dual-income household rate and the rate of minors per household (Table 2).

## 4. Discussion

Suicide mortality in Japan has been decreasing since 2009 [1,2,3]. The comprehensive regional suicide prevention programmes supported by governmental financial spending have played an important role in the improvement of suicide mortality in Japan [1,2,3]. However, these programmes predominantly reduced male suicide mortality, and had a limited contribution to female suicide mortality. It is well known that most women in Japan are traditionally forced to play subordinate roles because of the limited resources for childcare support and are prone to social isolation due to childcare demands [1,2]. A population-based study revealed that female suicide mortalities due to the reasons of ‘exhaustion from support for family member’, ‘caring for infirm family members’, and ‘raising children’ were statistically larger than that of male suicide mortalities in Japan [3,13]. Therefore, not only childcare, but also care of infirm family members is a major motive for suicide among Japanese females [3]. Based on these Japanese sociofamilial situations, we hypothesised that inadequate social/welfare support systems for childcare and caring for infirm family members in Japan impede social advancement of females, resulting in a lack of improvement in social isolation of females [1,2,3]. The results of this study support our hypothesis, since the rise in the dual-income household rate shows reduced suicide mortalities, albeit limited, among working-age females (ranging from females in their 30s to 50s) and female suicide mortality caused by romance-related motives. These results suggest that dual-income households lead to a suppression of social isolation of women and enable the development of their economic as well as psychological independence, leading to reduced suicide mortality among working-age females. In addition to a rise in the dual-income household rate contributing to the reduction in suicide mortality of working-age females, the present study detected the existence of multiple counter-partners for female suicide mortality, such as suicide mortalities of working-age males (20–50s), the elderly (over 60 years old—both males and females), and school-age populations. The increasing impact of the dual-income household rate on suicide mortality was more widespread than our expectation, comparable to that of the complete unemployment rate.

It can be theorised that the increased suicide mortalities of the elderly population induced by the rising dual-income household rate indicate that the elderly, who were traditionally supported by working-age females, are facing the effects of declining support provided by the household environment. Gender-specific features of this way of thinking become resembling according to aging [27]. Elderly suicide has been associated with poor social integration and self-perception as a burden on others, since suicide might be recognised as a solution to personal and social decline [8,28]. Indeed, enlightenment and gatekeeper development programmes in the comprehensive regional suicide programmes in Japan promoted the improvement for suicide mortality of elderly populations [1,2]. Therefore, enhancing the regional social/welfare support resources for elderly populations may improve the isolation of the elderly in dual-income households. Therefore, based on this theory, the decrease in the suicide mortality of elderly females in one-person households and facilities with an increase in the certification rate of long-term care insurance can be understood. Conversely, the suppressive impact of long-term care insurance on suicide mortality of elderly populations was found to be limited, less than our expectation, since the suicide mortality of only the females in their 80s decreased by the increase in the certification rate of long-term care insurance. This is caused by the different household structures in Japan. The elderly males in Japan primarily belong to the following households: ‘households with their spouse’ and ‘households with their spouse and descendants, but the elderly females primarily belong to the following households: ‘one-person households’ and ‘households such as facilities’ [26]. It has been well known that ‘one-person households’ is a risk factor for planning suicide [28,29]. The results show that long-term care insurance contributed to the reduction in suicide mortalities caused by health- and economic-related motives, suggesting that long-term care insurance might support not only the elderly individuals, but also their descendants who care for the elderly (i.e., certification rate of long-term care insurance was significantly/negatively related to male suicide mortalities caused by health-, economy- and school-related motives). In Japan, the impacts of mental illness containing major depression on suicide mortality predominantly affected the suicide motive of elderly females rather than physical illness [3,13]. Recent clinical studies reported that depressed patients with suicide ideation can be improved as almost good outcomes compared with depressed patients without suicide ideation via enhancement of positive mental health using cognitive-behavioural therapy [30,31]. Therefore, suicide mortality of elderly females caused by health-related motives might be prevented by the external cooperation of regional primary care physicians, psychiatry specialists and staffs in long-term care insurance, via suicide prevention programmes, due to the guidance to help them access appropriate services [3].

Previous studies detected that regional comprehensive suicide prevention programmes could not provide effective support for suicide mortality of school-age populations [1,2,3]. Surprisingly, municipal telephone consultation support programmes in regional suicide prevention programmes were positively related to female suicide mortalities of the school-age population and caused by school-related motives [3]. Telephone consultation programmes (also known as helplines, hotlines, crisis lines, etc.) are widely implemented to provide support for various psychosocial concerns among the youth because of their associated advantages such as confidential and accessible formats (through telephone, e-mail, social networking services, or chatrooms) [3,32,33]. According to these studies, currently, to increase suicide prevention among the youth, it is recommended to develop Internet consultation support programmes to accommodate the unique needs of young people in Japan [34]. Considering the surprising responses of suicide-prone school-age populations to telephone consultation programmes in Japan, the results of this study strongly indicate the importance of familial support that plays a fundamental role in the psychosocial development of young individuals [35]. The other significant effort that will assist in the suicide prevention efforts of school-age populations (specifically 10s) is increasing the rate of minors per household to reduce suicide mortalities caused by school-related motives. As parents cannot dedicate sufficient time to childcare in dual-income households, siblings complement the reduced familial communication by communicating with each other. Therefore, providing a combination of confidential support and affectionate family support is necessary for young people with serious distress. In other words, the development of confidential Internet support programmes alone cannot adequately address the serious distress among young individuals induced by isolation and reduced family communication in dual-income households, as such circumstances also negatively impact the psychosocial development of young individuals, leading to a requirement of additional support for such individuals.

This study detected a part of the underlying cause of suicide of young carers. Initially, the mechanisms by which the certification rate of long-term care insurance significantly/negatively affects male suicide mortality caused by school-related motives were difficult to discern. The MHLW reported in the ‘Survey Study on the Actual Conditions of Young Carers’ that approximately 4–6% of the school-age population might be candidate young carers [36]. According to the data of the ‘Comprehensive Survey of Living Conditions’ [37], the household structure of young carers in cities with a population lower than 150,000 was a three-generation household, in which young carers lived with their parents and their grandparents. Considering that the role of Japanese young carers has recently been focused upon, these young carers undertake some of the burden of care for grandparents and infirm family members, consequently suffering from the psychosocial effects of such a burden, resulting in their isolation. Therefore, the enrichment of long-term care insurance contributes to the reduction in distress in not only the elderly and working-age populations but also in that of young school-age carers. However, the increase in the elderly population in Japan not only increased the burden on school-age and working-age populations, it also contributed to the reduction in suicide mortality of individuals in their 20s and female suicide mortalities caused by family- and health-related motives. These results indicate the elderly individuals, who belong in a ‘household with a spouse and descendants’, can complement the vulnerabilities of Japanese families in the modern age and the resultant declining support for school-age children in dual-income households.

An increase in suicide mortalities of working-age male populations in Japan due to the rise in dual-income households suggests the seriousness of the work–life or work–family conflicts of Japanese working-age male populations. Work–family conflict is recognised as ‘a form of inter-role conflict in which the demands of work and family roles are incompatible in some respect so that participation in either the work or family role is more difficult because of participation in the other role’ [38]. Greenhaus and Powell have proposed an answer to this problem, designating it as work–family enrichment, which is considered as ‘the extent to which experience in one role improves the quality of the life in the other role’ [39]. However, Japanese society has yet to adopt the concept of work–family enrichment and associated good practices that are essential for the enlightenment of work–family enrichment. This problem might be synonymous with the lack of good practices regarding the achievement of enrichment between familial psychodynamics and the reduction in national power due to the reduced birth rate and ageing population. The family environment and familial psychodynamics are composed of diverse factors, such as values, personality, economy, construction and infirm family members. Thus, as compared to enhancement in familial support, the enhancement of the workplace support system is more likely, since the support programmes in the organisation are provided with a clear purpose for improving work efficiency and business performance (achieving goals) [40]. Furthermore, several studies reported that supervisors and colleagues may thrive via formal and/or informal support in workplace communities [41,42]. Based on previous findings, providing training opportunities to employees leads to improvement of mutual support among the colleagues, leading to indirectly improving the positive implications associated with family supportive culture and fostering good workplace practices.

Securing human resources who will form the future labour force and contribute to the socioeconomic development of the nation is one of the most important issues for Japanese society. Therefore, it is expected that the enhancement of the economic and social participation and contribution of women will likely contribute to the socioeconomic development in Japan. To counter the serious socioeconomic problems in Japan, the Gender Equality Bureau Cabinet Office has enacted the ‘Fifth Basic Plan for Gender Equality’ to pursue the promotion of social participation and contribution of females in Japan [16]. This basic plan is composed of three major factors of work–family culture: work–family organisational support, career consequences related to the use of work–family programmes, and workplace expectations hindering the fulfilment of the family roles of employees [15]. As mentioned in this study, working-age females have traditionally supported families and society in Japan, and the role played by working-age females will continue to be fundamental in maintaining the well-being of Japanese society. The Japanese sociofamilial situation may have inhibited the effects of comprehensive suicide prevention programmes targeted females [1,2,3]. However, dual-income families have suffered several issues in terms of conciliation between the working environment, the family context and the management of children and infirm family members [17]. This is the major theme in overcoming the issues relating to work–life and work–family balances and enrichments [43]. Therefore, it should be assumed that the rapid promotion of women’s social participation and contribution will cause disturbances via the progression of vulnerability of the immature social/welfare system associated with work–family balance in Japan. To promote women’s participation in society, there is a need to promote the concepts of work–life and work–family balances.

This study had several limitations. The present study adopted covariates such as household structural and social and employment factors; however, the social welfare, medical, and educational resource factors were not included in this study. These factors may provide additional detailed information regarding the impacts of dual-income households on suicide mortalities of school-age and elderly populations. It has been well established that a lower grade of education is a risk of suicidal behaviour [29,44], except for suicide attempts in that being female studying at university is a risk factor for a suicide attempt [45]. In the present study, complete unemployment rate is the most predominant impact factor against suicide mortality. Therefore, exploring the interaction between the educational backgrounds and employment status on suicide mortality probably clarify the fundamental information associated with suicide behaviour. Risks of suicide were 3.3 and 18.32 times higher among individuals who are widowers or separated/divorced, respectively [46]. Furthermore, the percentages decreases when individuals live in couple with children for childcare [47]. Therefore, marriage is considered to be a protective factor for suicide in elderly populations. In other words, the present study possibly revealed the presence of risk factors among suicide protection factors associated with household and life–work balance. Other Asian countries, such as China and South Korea, are likely to face the problem associated with a declining birth rate and an ageing populations, following Japan [48,49,50]. The recent ‘Global Gender Gap Report 2021’ mentioned that there is a need for enhancing the economic and social participation and contribution of women in Japan, China, and South Korea [51]. Furthermore, these three Asian countries achieved the dubious distinction of having the highest suicide mortality among the elderly population in the world [52]. In order to clarify the impact of the declining birth rate and ageing population on society, we aim to further report in more detail the impact of women’s social advancement, familial psychodynamics, and social and welfare resources on suicide mortality as compared with China and South Korea in the future.

## 5. Conclusions

In conclusion, the present study revealed that the rise in dual-income households contributes to the reduction in suicide mortality of working-age females, but possibly increases suicide mortalities of working-age males, school-age and elderly populations (comparable to the complete unemployment rate) due to declining support by working-age females in Japan. In Japanese society, the empowerment of women is a promising solution to supplement the lack of employment ability among the population due to the declining population. Furthermore, the identified findings in the present study suggest enhancement of social advancement of women seems to develop the independence of working-age female populations resulting in contribution to reducing the suicide mortality of the working-age female population in Japan. Conversely, the lack of key protective personnel in the households probably exerts a negative impact on school-aging, elderly individuals and working-age males. Therefore, to avoid the resulting sociofamilial disturbance, there is a need to develop the independence of Japanese individuals and enhance support resources for care for children, the elderly, and disabled persons.

## Figures and Tables

**Table 1:** Impacts of dual-income household rate (Model_1), household structural and social/employment factors (Model_2) on standardised death rates for suicide mortalities (SDR) of Male + Female, Male and Female between 2009 and 2017.

Factors	Male + Female			Male			Female		
	β	SE	*p* value	β	SE	*p* value	β	SE	*p* value
Model_1									
dual-income household rate	0.158	0.069	0.017 **	0.169	0.110	0.009 **	0.094	0.055	0.274
Model_2									
dual-income household rate	0.405	0.076	0.000 **	0.399	0.108	0.000 **	0.363	0.058	0.000 **
minors rate per household	0.044	0.034	0.126	0.035	0.058	0.236	0.051	0.025	0.213
elderly rate per household	−0.057	0.056	0.070	−0.038	0.091	0.238	−0.091	0.044	0.020 *
savings per household	−0.042	0.000	0.176	−0.073	0.001	0.034 *	0.033	0.000	0.286
liabilities per household	0.033	0.002	0.373	0.052	0.004	0.171	0.025	0.000	0.527
yearly incomes per household	−0.051	0.001	0.049 *	−0.082	0.001	0.006 **	−0.004	0.002	0.912
employment rate	−0.087	0.118	0.231	−0.106	0.191	0.202	−0.015	0.080	0.491
male temporary employment rate	−0.033	0.331	0.520	−0.045	0.581	0.477	−0.017	0.227	0.768
female temporary employment rate	−0.104	0.163	0.055	−0.101	0.286	0.107	−0.096	0.094	0.066
long-term care insurance rate	−0.155	0.015	0.034 *	−0.122	0.022	0.071	−0.062	0.010	0.383
complete unemployment rate	0.719	0.199	0.000 **	0.665	0.286	0.000 **	0.704	0.156	0.000 **

* *p* < 0.05 and ** *p* < 0.01 by the hierarchal linear regression model. SE: standard error. Blue and red pained fonts indicate decreasing and increasing factors of suicide mortality, respectively.

**Table 2:** Impacts of dual-income household rate and household structural factors (Model_3) on suicide mortalities of Male + Female, Male and Female in their 10s and caused by school-related motive between 2009 and 2017.

**10s**									
Factors	Male + Female			Male			Female		
Model_3	β	SE	*p* value	β	SE	*p* value	β	SE	*p* value
dual-income household rate	0.055	0.020	0.321	0.103	0.028	0.117	−0.056	0.019	0.720
minors rate per household	−0.185	0.022	0.008 *	−0.147	0.040	0.063	−0.136	0.017	0.007 *
elderly rate per household	−0.120	0.035	0.160	−0.151	0.057	0.061	0.004	0.035	0.839
savings per household	−0.036	0.000	0.672	0.011	0.000	0.882	−0.078	0.000	0.287
liabilities per household	−0.027	0.000	0.713	−0.016	0.001	0.816	−0.026	0.000	0.674
yearly incomes per household	0.072	0.001	0.453	0.065	0.002	0.423	0.080	0.001	0.369
**School-related motive**									
Factors	Male + Female			Male			Female		
Model_3	β	SE	*p* value	β	SE	*p* value	β	SE	*p* value
dual-income household rate	0.175	0.004	0.039 *	0.120	0.005	0.164	0.135	0.002	0.012 *
minors rate per household	−0.117	0.003	0.034 *	0.024	0.006	0.724	−0.108	0.002	0.023 *
elderly rate per household	−0.111	0.005	0.092	0.032	0.007	0.495	−0.093	0.006	0.229
savings per household	0.112	0.000	0.131	0.196	0.000	0.057	−0.049	0.000	0.298
liabilities per household	0.044	0.000	0.416	0.087	0.000	0.129	0.152	0.000	0.071
yearly incomes per household	0.073	0.000	0.334	−0.071	0.000	0.461	−0.034	0.000	0.659

* *p* < 0.05 by hierarchal linear regression model. Blue and red pained fonts indicate decreasing and increasing factors of suicide mortality, respectively.

## Data Availability

All data relevant to the study are included in the article or uploaded as supplementary information. All raw data are available to any persons from Japanese National databases in the Statistics Bureau of the Ministry of Internal Affairs and Communications (SBMIAC), Cabinet Office (CAO) and Ministry of Health, Labour and Welfare (MHLW).

## Supplementary Materials

The following are available online at https://www.mdpi.com/article/10.3390/ijerph18115670/s1, Supplementary Table S1: The summary of descriptive statistics of suicide mortalities desegregated by gender- age- and motive-factors between 2008 and 2017 (dependent variables). Supplementary Table S2: Effects of the execution amount of EFECBSC sub-divisions, unemployment rate and GDP per capita on EBSMR trends of male plus female, male and female. Supplementary Table S3: Impacts of dual-income household rate, other household and social/employment factors on suicide mortalities of Male+Female caused by motives associated with family-, health-, economy-, employment-, romance- and school-related problems between 2009 and 2017. Supplementary Table S4: Impacts of dual-income household rate, other household and social factors on suicide mortalities of Male caused by motives associated with family-, health-, economy-, employment-, romance- and school-related problems between 2009 and 2017. Supplementary Table S5: Impacts of dual-income household rate, other household and social factors on suicide mortalities of Female caused by motives associated with family-, health-, economy-, employment-, romance- and school-related problems between 2009 and 2017. Supplementary Table S6: Impacts of dual-income household rate, other household and social factors on age-dependent suicide mortalities of Male+Female between 2009 and 2017. Supplementary Table S7: Impacts of dual-income household rate, other household and social factors on age-dependent suicide mortalities of Male between 2009 and 2017. Supplementary Table S8: Impacts of dual-income household rate, other household and social factors on age-dependent suicide mortalities of Female between 2009 and 2017.
