# Supplementary material for: Impacts of Dual-Income Household Rate on Suicide Mortalities in Japan
Source: Int J Environ Res Public Health. 2021 May 25;18(11):5670. doi: 10.3390/ijerph18115670 (PMC8199206; doi:10.3390/ijerph18115670)
Supplement: Supplementary file 1 [file ijerph-18-05670-s001.zip › ijerph-1234492-supplementary.pdf]

Supplemental Data

Supplementary Table 1: The summary of descriptive statistics of suicide mortalities desegregated by gender- age- and motive-factors between 2008 and 2017 (dependent variables). (p2).

Supplementary Table 2: Effects of the execution amount of EFECBSC sub-divisions, unemployment rate and GDP per capita on EBSMR trends of male plus female, male and female. (p2).

Supplementary Table 3: Impacts of dual-income household rate, other household and social/employment factors on suicide mortalities of Male+Female caused by motives associated with family-, health-, economy-, employment-, romance- and school-related problems between 2009 and 2017. (p3).

Supplementary Table 4: Impacts of dual-income household rate, other household and social factors on suicide mortalities of Male caused by motives associated with family-, health-, economy-, employment-, romance- and school-related problems between 2009 and 2017. (p3).

Supplementary Table 5: Impacts of dual-income household rate, other household and social factors on suicide mortalities of Female caused by motives associated with family-, health-, economy-, employment-, romance- and school-related problems between 2009 and 2017. (p4).

Supplementary Table 6: Impacts of dual-income household rate, other household and social factors on age-dependent suicide mortalities of Male+Female between 2009 and 2017. (p4).

Supplementary Table 7: Impacts of dual-income household rate, other household and social factors on age-dependent suicide mortalities of Male between 2009 and 2017. (p5).

Supplementary Table 8: Impacts of dual-income household rate, other household and social factors on age-dependent suicide mortalities of Female between 2009 and 2017. (p5).

Supplementary Table S1: The summary of descriptive statistics of suicide mortalities desegregated by gender- age- and motive-factors between 2009 and 2017 (dependent variables).

	Male+Female			Male			Female		
	mean	±	SD	mean	±	SD	mean	±	SD
(Age)									
SDR per population (100,000)	21.25	±	4.20	30.74	±	6.51	12.32	±	2.61
10s	2.40	±	1.03	3.22	±	1.67	1.54	±	1.11
20s	20.45	±	4.92	29.36	±	7.68	11.17	±	4.52
30s	21.87	±	5.20	30.28	±	7.60	10.96	±	4.04
40s	25.81	±	6.54	36.22	±	10.25	12.20	±	4.25
50s	29.71	±	7.41	41.88	±	11.74	14.17	±	4.34
60s	26.23	±	6.85	34.92	±	10.09	14.34	±	4.48
70s	26.68	±	6.24	35.71	±	9.80	17.57	±	5.85
80s	28.33	±	8.20	44.20	±	13.53	18.20	±	7.50
(Motive)									
Family	3.22	±	1.00	4.33	±	1.50	2.20	±	0.81
Health	10.69	±	3.06	13.05	±	4.06	8.49	±	2.49
Economy	4.19	±	1.87	7.79	±	3.56	0.82	±	0.45
Employment	1.86	±	0.61	3.34	±	1.17	0.38	±	0.25
Romance	0.73	±	0.29	0.93	±	0.45	0.50	±	0.31
School	0.28	±	0.18	0.43	±	0.30	0.14	±	0.16

Supplementary Table S2: The summary of descriptive statistics of household and social/employment factors and their VIF (Variance Inflation Factor) between 2009 and 2017 (independent variables and covariates).

	mean	±	SD	VIF
(Household factor)				
dual-income household rate (%)	26.71	±	3.98	2.62
minority rate per household	0.29	±	0.03	1.62
elderly rate per household	0.06	±	0.02	2.07
savings per household (million YEN)	11.98	±	3.09	2.55
liabilities per household (million YEN)	6.71	±	1.78	1.45
yearly incomes per household (million YEN)	6.96	±	0.69	3.26
(Social/employment factor)				
employment rate (%)	43.01	±	2.83	2.49
male temporary employment rate (%)	2.56	±	0.72	3.01
female temporary employment rate (%)	7.24	±	1.44	2.84
certification of long-term Care Insurance ratio per population (100,000)	185.25	±	19.72	1.85
completely unemployment rate (%)	3.68	±	1.06	2.20

Supplementary Table S3: Impacts of dual-income household rate, other household and social/employment factors on suicide mortalities of Male+Female caused by motives associated with family-, health-, economy-, employment-, romance- and school-related problems between 2009 and 2017.

Factors	family				health				economy				employment				romance				school			
	β	SE	P value		β	SE	P value		β	SE	P value		β	SE	P value		β	SE	P value		β	SE	P value	
Model_1																								
dual-income rate	0.056	0.022	0.013	*	0.103	0.087	0.243		0.039	0.033	0.243		0.034	0.013	0.015	*	-0.006	0.005	0.193		0.007	0.003	0.068	
X^2 value	130.70		<0.001	**	240.78		<0.001	**	132.45		<0.001	**	118.22		<0.001	**	40.33		<0.001	**	48.03		<0.001	**
Model_2																								
dual-income rate	0.093	0.025	0.000	**	0.270	0.095	0.007	**	0.140	0.036	0.000	**	0.049	0.017	0.006	**	0.005	0.006	0.427		0.009	0.004	0.023	*
minority rate	0.011	0.016	0.475		0.036	0.037	0.321		0.045	0.023	0.049	*	0.005	0.009	0.607		-0.001	0.005	0.782		-0.006	0.003	0.042	*
elderly rate	-0.029	0.026	0.262		-0.022	0.044	0.614		0.048	0.035	0.161		-0.015	0.012	0.211		-0.022	0.009	0.011	*	-0.007	0.005	0.147	
savings	0.000	0.000	0.487		0.000	0.000	0.761		0.000	0.000	0.354		0.000	0.000	0.534		0.000	0.000	0.919		0.000	0.000	0.132	
liabilities	-0.002	0.001	0.109		0.000	0.002	0.847		0.003	0.002	0.101		0.000	0.001	0.714		0.000	0.000	0.967		0.000	0.000	0.525	
yearly incomes	0.000	0.000	0.957		-0.001	0.001	0.079		-0.001	0.000	0.006	**	0.000	0.000	0.355		0.000	0.000	0.118		0.000	0.000	0.463	
employment rate	-0.028	0.036	0.439		-0.362	0.111	0.001	**	-0.130	0.061	0.034	*	-0.004	0.024	0.864		0.000	0.011	0.975		-0.003	0.005	0.512	
male temporary employment rate	0.102	0.147	0.486		0.304	0.328	0.354		0.160	0.271	0.556		0.073	0.071	0.307		0.015	0.046	0.746		0.022	0.027	0.405	
female temporary employment rate	-0.117	0.067	0.082		-0.062	0.157	0.695		-0.225	0.095	0.018	*	-0.060	0.035	0.081		-0.018	0.022	0.408		-0.001	0.013	0.933	
long-term Care Insurance	0.000	0.005	0.926		-0.041	0.015	0.006	**	-0.039	0.009	0.000	**	-0.002	0.002	0.314		-0.001	0.001	0.369		-0.001	0.001	0.124	
complete unemployment rate	0.307	0.069	0.000	**	1.276	0.205	0.000	**	1.091	0.094	0.000	**	0.142	0.046	0.002	**	0.073	0.028	0.008	**	0.014	0.011	0.206	
X^2 value	212.45		<0.001	**	443.467		<0.001	**	445.867		<0.001	**	160.30		<0.001	**	89.121		<0.001	**	67.518		<0.001	**

*:P<0.05 and **P<0.01 by hierarchal linear regression model analysis.

Supplementary Table S4: Impacts of dual-income household rate, other household and social factors on suicide mortalities of Male caused by motives associated with family-, health-, economy-, employment-, romance- and school-related problems between 2009 and 2017.

Factors	family				health				economy				employment				romance				school			
	β	SE	P value		β	SE	P value		β	SE	P value		β	SE	P value		β	SE	P value		β	SE	P value	
Model_1																								
dual-income rate	0.097	0.032	0.004	**	0.138	0.110	0.214		0.081	0.062	0.193		0.062	0.022	0.008	**	0.007	0.009	0.456		0.009	0.005	0.121	
X^2 value	127.52		<0.001	**	231.92		<0.001	**	131.65		<0.001	**	107.60		<0.001	**	35.77		<0.001	**	39.61		<0.001	**
Model_2																								
dual-income rate	0.139	0.036	0.000	**	0.310	0.124	0.016	*	0.266	0.064	0.000	**	0.084	0.030	0.007	**	0.012	0.010	0.261		0.011	0.006	0.073	
minority rate	0.024	0.026	0.352		0.058	0.053	0.275		0.082	0.044	0.062		0.028	0.027	0.307		0.007	0.009	0.463		0.002	0.006	0.761	
elderly rate	-0.003	0.037	0.939		0.037	0.070	0.601		0.101	0.066	0.126		0.035	0.027	0.197		-0.004	0.015	0.781		0.006	0.007	0.418	
savings	-0.001	0.001	0.311		0.001	0.003	0.800		0.005	0.003	0.114		0.000	0.001	0.830		-0.001	0.001	0.248		0.000	0.000	0.283	
liabilities	0.000	0.000	0.640		-0.001	0.001	0.415		-0.001	0.001	0.262		0.000	0.000	0.399		0.000	0.000	0.859		0.000	0.000	0.042	*
yearly incomes	-0.001	0.000	0.082		-0.002	0.001	0.070		-0.002	0.001	0.011	*	0.000	0.000	0.415		0.000	0.000	0.043	*	0.000	0.000	0.124	
employment rate	-0.082	0.054	0.130		-0.496	0.157	0.002	**	-0.264	0.118	0.025	*	-0.031	0.048	0.519		0.006	0.015	0.680		-0.008	0.008	0.369	
male temporary employment rate	0.163	0.234	0.487		0.249	0.503	0.620		0.342	0.517	0.508		0.048	0.134	0.720		-0.009	0.055	0.870		0.075	0.050	0.136	
female temporary employment rate	-0.135	0.105	0.200		-0.014	0.251	0.955		-0.430	0.184	0.020	*	-0.082	0.068	0.230		-0.005	0.026	0.840		-0.028	0.017	0.101	
long-term Care Insurance	-0.006	0.007	0.404		-0.055	0.020	0.006	**	-0.074	0.017	0.000	**	-0.008	0.006	0.146		-0.001	0.001	0.333		-0.003	0.001	0.000	**
complete unemployment rate	0.392	0.110	0.000	**	1.396	0.297	0.000	**	2.058	0.194	0.000	**	0.272	0.083	0.001	**	0.103	0.032	0.002	**	0.026	0.020	0.202	
X^2 value	202.97		<0.001	**	116.491		<0.001	**	440.071		<0.001	**	150.10		<0.001	**	74.487		<0.001	**	62.779		<0.001	**

*:P<0.05 and **P<0.01 by hierarchal linear regression model analysis.

Supplementary Table S5: Impacts of dual-income household rate, other household and social factors on suicide mortalities of Female caused by motives associated with family-, health-, economy-, employment-, romance- and school-related problems between 2009 and 2017.

Factors	family				health				economy				employment				romance				school		
	β	SE	P value		β	SE	P value		β	SE	P value		β	SE	P value		β	SE	P value		β	SE	P value
Model_1																							
dual-income rate	0.012	0.015	0.416		0.069	0.068	0.317		0.001	0.008	0.940		0.002	0.003	0.581		-0.014	0.004	0.001	**	0.005	0.002	0.019
X^2 value	61.14		<0.001	**	191.20		<0.001	**	51.67		<0.001	**	5.29		0.382		23.28		<0.001	**	6.13		0.294
Model_2																							
dual-income rate	0.046	0.017	0.009	**	0.233	0.070	0.002	**	0.021	0.009	0.024	*	0.006	0.005	0.210		-0.006	0.005	0.196		0.006	0.003	0.016
minority rate	-0.001	0.014	0.927		0.012	0.027	0.641		0.005	0.007	0.473		-0.005	0.004	0.205		0.003	0.006	0.605		-0.006	0.002	0.015
elderly rate	-0.057	0.024	0.018	*	-0.091	0.044	0.037	*	-0.006	0.011	0.560		-0.006	0.009	0.494		-0.016	0.009	0.087		-0.008	0.006	0.173
savings	0.001	0.000	0.054		-0.001	0.001	0.313		0.000	0.000	0.086		0.000	0.000	0.864		0.000	0.000	0.027	*	0.000	0.000	0.376
liabilities	0.000	0.000	0.033	*	0.000	0.000	0.481		0.000	0.000	0.983		0.000	0.000	0.863		0.000	0.000	0.317		0.000	0.000	0.069
yearly incomes	-0.002	0.001	0.093		0.000	0.002	0.872		0.001	0.001	0.314		0.000	0.000	0.768		0.000	0.000	0.780		0.000	0.000	0.525
employment rate	0.021	0.025	0.411		-0.178	0.084	0.034	*	-0.005	0.013	0.711		-0.002	0.006	0.754		-0.004	0.011	0.736		0.002	0.005	0.681
male temporary employment rate	0.050	0.112	0.654		0.368	0.249	0.141		0.006	0.062	0.917		0.044	0.029	0.129		0.033	0.044	0.460		0.028	0.021	0.175
female temporary employment rate	-0.106	0.054	0.051		-0.111	0.098	0.258		-0.040	0.027	0.134		-0.029	0.013	0.019		-0.039	0.019	0.042	*	-0.016	0.010	0.111
long-term Care Insurance	0.002	0.003	0.451		-0.026	0.011	0.021	*	-0.002	0.002	0.271		-0.002	0.001	0.154		-0.002	0.001	0.168		0.000	0.001	0.864
complete unemployment rate	0.200	0.053	0.000	**	1.191	0.152	0.000	**	0.180	0.024	0.000	**	0.026	0.019	0.178		0.044	0.027	0.098		0.004	0.010	0.691
X^2 value	107.67		<0.001	**	369.365		<0.001	**	135.276		<0.001	**	18.19		0.253		59.876		<0.001	**	21.611		0.118

*:P<0.05 and **P<0.01 by hierarchical linear regression model analysis.

Supplementary Table S6: Impacts of dual-income household rate, other household and social factors on age-dependent suicide mortalities of Male+Female between 2009 and 2017.

Factors	10s				20s				30s				40s			
	β	SE	P value		β	SE	P value		β	SE	P value		β	SE	P value	
Model_1																
dual-income rate	0.012	0.020	0.566		0.172	0.088	0.056		0.155	0.093	0.103		0.184	0.101	0.075	
X^2 value	21.58		0.001	*	47.63		<0.001	**	100.26		<0.001	**	93.83		<0.001	**
Model_2																
dual-income rate	0.010	0.023	0.682		0.426	0.099	0.000	**	0.326	0.101	0.002	**	0.683	0.121	0.000	**
minority rate	-0.059	0.022	0.009	**	-0.128	0.072	0.074		0.122	0.059	0.038	*	0.019	0.082	0.816	
elderly rate	-0.058	0.036	0.103		-0.295	0.112	0.009	**	0.015	0.116	0.900		-0.235	0.127	0.065	
savings	0.000	0.000	0.730		0.000	0.001	0.823		-0.002	0.001	0.005	**	-0.001	0.001	0.622	
liabilities	0.000	0.001	0.981		0.004	0.005	0.374		0.008	0.004	0.052		-0.004	0.005	0.428	
yearly incomes	0.000	0.000	0.735		0.000	0.002	0.812		-0.003	0.001	0.025	*	0.000	0.001	0.833	
employment rate	0.048	0.033	0.149		0.217	0.137	0.113		0.031	0.111	0.782		0.085	0.178	0.634	
male temporary employment rate	0.048	0.157	0.759		-0.288	0.608	0.636		-0.133	0.581	0.819		0.756	0.642	0.240	
female temporary employment rate	-0.078	0.065	0.233		-0.563	0.319	0.078		-0.653	0.292	0.026	*	-0.923	0.375	0.014	*
long-term Care Insurance	-0.002	0.004	0.614		0.001	0.018	0.964		-0.019	0.016	0.227		-0.030	0.024	0.201	
complete unemployment rate	-0.001	0.083	0.991		2.310	0.385	0.000	**	2.322	0.333	0.000	**	4.220	0.420	0.000	**
X^2 value	36.26		0.002		152.952		<0.001	**	253.543		<0.001	**	325.36		<0.001	**

Factors	50s				60s				70s				80s			
	β	SE	P value		β	SE	P value		β	SE	P value		β	SE	P value	
Model_1																
dual-income rate	0.043	0.123	0.726		0.108	0.092	0.244		0.307	0.095	0.002	**	0.706	0.148	0.000	**
X^2 value	119.95		<0.001	**	140.27		<0.001	**	133.18		<0.001	**	271.48		<0.001	**
Model_2																
dual-income rate	0.536	0.102	0.000	**	0.592	0.118	0.000	**	0.653	0.134	0.000	**	0.891	0.203	0.000	**
minority rate	0.163	0.105	0.123		0.115	0.082	0.160		0.070	0.088	0.426		-0.049	0.092	0.594	
elderly rate	-0.213	0.154	0.167		-0.053	0.110	0.632		-0.070	0.147	0.637		0.011	0.139	0.934	
savings	-0.001	0.001	0.422		-0.002	0.001	0.112		0.000	0.001	0.858		0.001	0.001	0.571	
liabilities	0.002	0.006	0.741		0.006	0.006	0.272		0.006	0.006	0.342		0.000	0.006	0.984	
yearly incomes	-0.002	0.002	0.227		-0.001	0.001	0.391		-0.002	0.002	0.221		-0.002	0.002	0.341	
employment rate	0.059	0.232	0.800		-0.033	0.257	0.899		0.039	0.166	0.812		-0.356	0.274	0.195	
male temporary employment rate	-1.378	0.700	0.050	*	0.010	0.842	0.991		-0.383	0.663	0.564		-0.727	0.648	0.262	
female temporary employment rate	0.158	0.329	0.632		-0.355	0.403	0.378		0.125	0.369	0.735		-0.303	0.403	0.452	
long-term Care Insurance	-0.004	0.030	0.883		-0.019	0.024	0.421		0.037	0.028	0.180		-0.027	0.029	0.346	
complete unemployment rate	4.603	0.382	0.000	**	4.972	0.407	0.000	**	3.152	0.478	0.000	**	2.368	0.471	0.000	**
X^2 value	360.164		<0.001	**	457.406		<0.001	**	248.05		<0.001	**	364.238		<0.001	**

*:P<0.05 and **P<0.01 by hierarchical linear regression model analysis.

Supplementary Table S7: Impacts of dual-income household rate, other household and social factors on age-dependent suicide mortalities of Male between 2009 and 2017.

Factors	10s				20s				30s				40s				50s				60s				70s				80s			
	β	SE	P value		β	SE	P value		β	SE	P value		β	SE	P value		β	SE	P value		β	SE	P value		β	SE	P value		β	SE	P value	
Model_1																																
dual-income rate	0.022	0.027	0.415		0.390	0.139	0.007	**	0.250	0.152	0.107		0.352	0.160	0.033	*	0.258	0.181	0.162		0.061	0.165	0.713		0.205	0.152	0.184		0.779	0.246	0.003	**
X^2 value	10.09		0.073		50.55		<0.001	**	61.41		<0.001	**	104.57		<0.001	**	86.08		<0.001	**	119.32		<0.001	**	88.52		<0.001	**	126.76		<0.001	**
Model_2																																
dual-income rate	0.033	0.034	0.327		0.716	0.142	0.000	**	0.363	0.155	0.024	*	0.840	0.173	0.000	**	0.863	0.156	0.000	**	0.694	0.161	0.000	**	0.608	0.159	0.000	**	1.203	0.257	0.000	**
minority rate	-0.075	0.031	0.018		-0.210	0.128	0.100		-0.057	0.110	0.604		0.119	0.149	0.426		0.180	0.151	0.236		0.143	0.110	0.197		0.161	0.163	0.322		0.193	0.170	0.257	
elderly rate	-0.107	0.051	0.035		-0.561	0.180	0.002	**	0.098	0.184	0.595		-0.151	0.238	0.526		-0.416	0.281	0.140		0.004	0.213	0.985		0.027	0.199	0.891		-0.124	0.247	0.616	
savings	0.000	0.000	0.741		0.001	0.002	0.552		-0.001	0.002	0.547		-0.006	0.002	0.004	**	-0.005	0.002	0.009	**	-0.004	0.002	0.010	**	-0.003	0.002	0.195		-0.004	0.003	0.228	
liabilities	0.001	0.002	0.698		0.000	0.008	0.972		-0.007	0.007	0.306		0.024	0.009	0.006	**	0.022	0.010	0.036	*	0.008	0.007	0.255		0.000	0.009	0.997		-0.008	0.013	0.519	
yearly incomes	0.000	0.001	0.946		0.000	0.002	0.900		-0.002	0.002	0.379		-0.007	0.003	0.025	*	-0.010	0.003	0.002	**	-0.006	0.002	0.019	*	-0.006	0.003	0.055		-0.003	0.004	0.401	
employment rate	0.043	0.054	0.428		0.326	0.248	0.189		0.249	0.194	0.200		0.156	0.252	0.537		0.131	0.338	0.698		-0.296	0.338	0.380		-0.178	0.297	0.549		-0.015	0.416	0.971	
male temporary employment rate	-0.019	0.222	0.933		-0.879	1.011	0.385		-2.192	0.938	0.020	*	-0.670	1.127	0.553		-2.849	1.541	0.065		-0.226	1.007	0.823		-1.044	1.193	0.382		-0.196	1.931	0.919	
female temporary employment rate	-0.114	0.110	0.298		-0.548	0.576	0.342		-0.145	0.479	0.762		-0.488	0.535	0.362		0.446	0.686	0.515		-0.492	0.508	0.333		0.160	0.653	0.807		-0.270	0.872	0.757	
long-term Care Insurance	-0.002	0.006	0.793		0.022	0.030	0.464		0.020	0.022	0.375		0.001	0.037	0.988		0.010	0.043	0.817		-0.056	0.037	0.126		0.015	0.045	0.742		0.083	0.054	0.122	
complete unemployment rate	0.012	0.123	0.923		2.655	0.562	0.000	**	2.426	0.557	0.000	**	5.598	0.506	0.000	**	6.133	0.652	0.000	**	6.427	0.577	0.000	**	4.244	0.592	0.000	**	3.232	0.909	0.000	**
X^2 value	23.04		0.083		116.491		<0.001	**	497.697		<0.001	**	286.04		<0.001	**	275.239		<0.001	**	401.321		<0.001	**	201.44		<0.001	**	174.275		<0.001	**

*:P<0.05 and **P<0.01 by hierarchal linear regression model analysis.

Supplementary Table S8: Impacts of dual-income household rate, other household and social factors on age-dependent suicide mortalities of Female between 2009 and 2017.

Factors	10s				20s				30s				40s				50s				60s				70s				80s			
	β	SE	P value		β	SE	P value		β	SE	P value		β	SE	P value		β	SE	P value		β	SE	P value		β	SE	P value		β	SE	P value	
Model_1																																
dual-income rate	-0.002	0.017	0.910		-0.119	0.069	0.089		-0.106	0.044	0.020	*	-0.158	0.059	0.010	**	-0.143	0.068	0.042	*	0.082	0.059	0.169		0.347	0.102	0.001	**	0.532	0.126	0.000	**
X^2 value	5.83		0.323		25.81		<0.001	**	55.00		<0.001	**	53.47		<0.001	**	33.36		<0.001	**	63.65		<0.001	**	97.30		<0.001	**	195.56		<0.001	**
Model_2																																
dual-income rate	-0.016	0.021	0.460		0.093	0.078	0.236		0.107	0.059	0.075		0.117	0.082	0.159		-0.009	0.075	0.910		0.325	0.081	0.000	**	0.695	0.186	0.001	**	0.748	0.179	0.000	**
minority rate	-0.047	0.017	0.005		-0.016	0.083	0.845		0.027	0.081	0.741		-0.153	0.069	0.027	*	0.014	0.089	0.877		-0.115	0.062	0.065		0.056	0.088	0.525		0.306	0.106	0.004	**
elderly rate	-0.010	0.035	0.773		0.007	0.134	0.958		-0.088	0.097	0.365		-0.243	0.112	0.031	*	0.016	0.148	0.916		-0.261	0.114	0.023	*	-0.206	0.164	0.209		-0.099	0.167	0.554	
savings	0.000	0.000	0.375		0.000	0.001	0.813		0.000	0.001	0.923		0.000	0.001	0.885		0.000	0.001	0.765		0.000	0.001	0.825		-0.001	0.002	0.616		-0.002	0.001	0.250	
liabilities	0.000	0.000	0.246		-0.001	0.001	0.492		0.001	0.001	0.514		0.002	0.001	0.027	*	0.000	0.001	0.772		-0.001	0.001	0.266		0.001	0.001	0.615		0.000	0.001	0.975	
yearly incomes	0.000	0.001	0.979		0.011	0.004	0.014	*	0.003	0.004	0.494		0.000	0.005	0.992		-0.003	0.005	0.513		0.002	0.005	0.699		-0.009	0.006	0.135		0.007	0.006	0.281	
employment rate	0.041	0.027	0.130		0.039	0.116	0.736		0.119	0.103	0.246		0.048	0.117	0.682		0.084	0.157	0.593		0.227	0.150	0.129		0.121	0.208	0.562		-0.432	0.243	0.077	
male temporary employment rate	0.098	0.129	0.448		0.212	0.493	0.668		-0.312	0.513	0.544		-0.046	0.485	0.925		-0.152	0.430	0.724		0.222	0.444	0.618		-0.615	0.709	0.386		0.054	0.638	0.933	
female temporary employment rate	-0.040	0.049	0.418		-0.437	0.265	0.100		0.087	0.206	0.672		-0.044	0.241	0.856		0.036	0.216	0.867		-0.325	0.249	0.193		0.303	0.409	0.459		-0.106	0.383	0.782	
long-term Care Insurance	-0.003	0.003	0.403		-0.007	0.014	0.597		-0.008	0.010	0.415		-0.012	0.015	0.438		0.002	0.016	0.888		-0.009	0.017	0.581		-0.016	0.030	0.600		-0.084	0.030	0.005	**
complete unemployment rate	-0.035	0.061	0.566		2.005	0.370	0.000	**	1.957	0.276	0.000	**	2.053	0.272	0.000	**	1.500	0.268	0.000	**	2.226	0.309	0.000	**	2.408	0.461	0.000	**	2.427	0.560	0.000	**
X^2 value	18.41		0.242		93.947		<0.001	**	131.279		<0.001	**	142.94		<0.001	**	74.926		<0.001	**	158.901		<0.001	**	171.42		<0.001	**	295.963		<0.001	**

*:P<0.05 and **P<0.01 by hierarchal linear regression model analysis.
